# Pathophysiological Association of Endothelial Dysfunction with Fatal Outcome in COVID-19

**DOI:** 10.3390/ijms22105131

**Published:** 2021-05-12

**Authors:** Tatsuya Maruhashi, Yukihito Higashi

**Affiliations:** 1 Department of Cardiovascular Regeneration and Medicine, Research Institute for Radiation Biology and Medicine, Hiroshima University, Hiroshima 734-8553, Japan; maru0512@hiroshima-u.ac.jp; 2 Division of Regeneration and Medicine, Medical Center for Translational and Clinical Research, Hiroshima University Hospital, Hiroshima 734-8551, Japan

**Keywords:** SARS-CoV-2, endothelial function, thromboinflammation, angiotensin-converting enzyme 2

## Abstract

The outbreak of coronavirus disease 2019 (COVID-19) caused by the betacoronavirus SARS-CoV-2 is now a worldwide challenge for healthcare systems. Although the leading cause of mortality in patients with COVID-19 is hypoxic respiratory failure due to viral pneumonia and acute respiratory distress syndrome, accumulating evidence has shown that the risk of thromboembolism is substantially high in patients with severe COVID-19 and that a thromboembolic event is another major complication contributing to the high morbidity and mortality in patients with COVID-19. Endothelial dysfunction is emerging as one of the main contributors to the pathogenesis of thromboembolic events in COVID-19. Endothelial dysfunction is usually referred to as reduced nitric oxide bioavailability. However, failures of the endothelium to control coagulation, inflammation, or permeability are also instances of endothelial dysfunction. Recent studies have indicated the possibility that SARS-CoV-2 can directly infect endothelial cells via the angiotensin-converting enzyme 2 pathway and that endothelial dysfunction caused by direct virus infection of endothelial cells may contribute to thrombotic complications and severe disease outcomes in patients with COVID-19. In this review, we summarize the current understanding of relationships between SARS-CoV-2 infection, endothelial dysfunction, and pulmonary and extrapulmonary complications in patients with COVID-19.

## 1. Introduction

**Figure 1:**
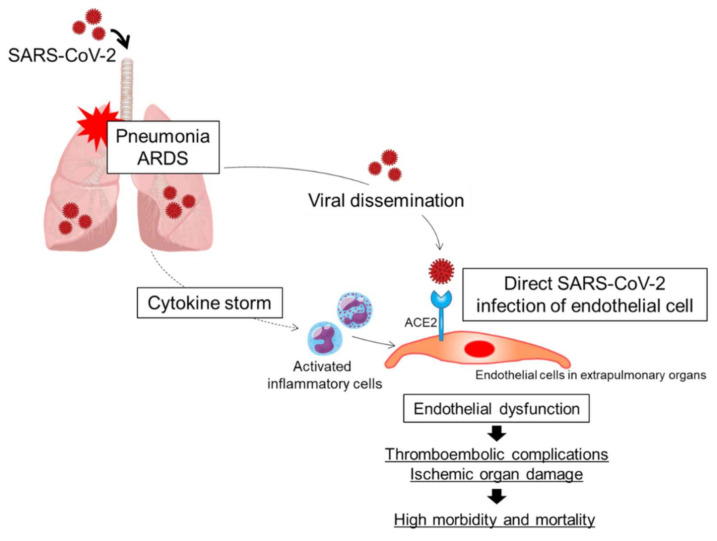
Putative mechanisms of endothelial dysfunction in patients with COVID-19. A combination of direct SARS-CoV-2 infection of endothelial cells and a cytokine storm originating in the lungs may synergistically cause endothelial cell injury and/or activation, leading to endothelial dysfunction. ARDS, acute respiratory distress syndrome; ACE2, angiotensin-converting enzyme 2.

The outbreak of coronavirus disease 2019 (COVID-19) caused by the betacoronavirus SARS-CoV-2 is now a worldwide challenge for healthcare systems. Common complications of severe COVID-19 are viral pneumonia and acute respiratory distress syndrome (ARDS). Although hypoxic respiratory failure due to viral pneumonia and ARDS is the leading cause of mortality in patients with COVID-19 [1], accumulating evidence has shown that the risk of venous thromboembolism, arterial thrombosis, and small-vessel thrombosis is substantially high and that a thromboembolic event is another major complication in patients with COVID-19, especially in critically ill patients in the intensive care unit, contributing to the high morbidity and mortality in patients with COVID-19 [2,3,4,5,6,7]. Although the pathogenesis of thrombotic complications in patients with severe COVID-19 is the focus of intensive investigation, the dysfunctional endothelium is emerging as one of the main contributors to the pathogenesis of thrombosis. Endothelial cells play pivotal roles in regulating the balances of vascular tone, coagulation, inflammation, and permeability. In patients with severe COVID-19, a combination of a cytokine storm originating in the lung and direct SARS-CoV-2 infection of endothelial cells may synergistically cause endothelial dysfunction, contributing to the thromboembolic complications through excessive coagulation and thromboinflammation in patients with severe COVID-19 (Figure 1). In this review, we summarize the current understanding of the mechanisms of endothelial dysfunction and the associations of endothelial dysfunction with pulmonary and extrapulmonary complications in COVID-19. First, we discuss the function of the healthy endothelium. Next, we discuss the possibility of direct SARS-CoV-2 infection of endothelial cells. Direct SARS-CoV-2 infection of endothelial cells may be one of the main mechanisms of endothelial dysfunction in COVID-19. Next, we discuss the mechanisms of downregulation of angiotensin-converting enzyme 2 (ACE2). Downregulation of ACE2 may be associated with endothelial dysfunction in COVID-19. Next, we discuss the association between thromboinflammation and endothelial dysfunction. Finally, we discuss the associations of endothelial dysfunction with pulmonary and extrapulmonary complications in patients with COVID-19.

## 2. Functions of Resting Endothelial Cells

The surface area of the endothelium monolayer that lines the intima of the whole circulatory system in the human body is estimated to measure up to 7000 m^2^ [8]. About 98% of endothelial cells are assumed to reside within the microvasculature, reflecting the vast surface area of the microcirculatory system in the human body. The vascular endothelium functions not only as a structural barrier that separates the blood vessel wall and the inside cavity but also as an endocrine organ that secretes various vasoactive agents such as nitric oxide (NO), prostacyclin, endothelium-derived hyperpolarizing factor, endothelin-1, angiotensin II, and thromboxane A_2_ [9]. NO has various antiatherosclerotic effects such as vasodilation, inhibition of platelet adhesion and aggregation, inhibition of leukocyte adhesion, and suppression of vascular smooth muscle cell proliferation [10,11]. The healthy endothelium acts as a gatekeeper controlling vascular homeostasis by regulating the moment-to-moment balance between vasodilation and vasoconstriction, antithrombosis and prothrombosis, anti-inflammation and proinflammation, antioxidation and pro-oxidation, and vascular smooth muscle cell growth inhibition and promotion [12]. Endothelial dysfunction is often referred to as a condition in which NO bioavailability is reduced due to decreased NO production from the endothelium and/or increased NO inactivation by reactive oxygen species [13]. Failures of the endothelium to control coagulation, permeability, or leukocyte quiescence are also instances of endothelial dysfunction [13].

The endothelium actively maintains blood fluidity by inhibiting blood coagulation and platelet activation [14]. In general, coagulation is initiated by tissue factors (TFs) expressed on the cell surface [15,16]. The TF/factor (F) VIIa complex on the TF-bearing cell surface activates a small amount of FX. The activated FX (FXa) and its cofactor FVa form prothrombinase complexes (FVa/FXa) on the TF-bearing cell surface, which convert prothrombin to thrombin. The small amount of thrombin produced by TF-bearing cells activates platelets and coagulation factors, leading to the concurrent formations of tenase complexes and FVa/FXa on the platelet surface. Tenase complexes activate FX, and FVa/FXa converts prothrombin to thrombin. Therefore, concurrent formations of tense complexes and FVa/FXa on the platelet surface synergistically upregulate thrombin generation. Once these enzyme complexes begin to work on the platelet surface, thrombin production rapidly increases, and the thrombin produced by platelets activates additional platelets and coagulation factors, leading to a burst of thrombin generation that is sufficient to form and stabilize fibrin clots.

**Figure 2:**
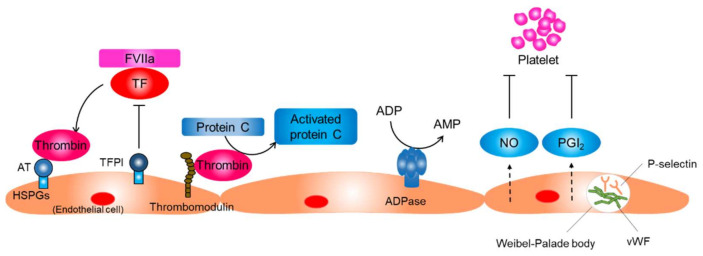
Function of the resting endothelium. The normal resting endothelium plays an important role in maintaining vascular homeostasis by producing several anti-inflammatory and antithrombotic factors. AT, antithrombin; HSPG, heparin sulfate proteoglycans; TF, tissue factor; TFPI, tissue factor pathway inhibitor; FVIIa, factor VIIa; NO, nitric oxide; PGI_2_, prostaglandin I_2_; vWF, von Willebrand factor.

The endothelium neutralizes thrombin activity through several mechanisms (Figure 2). Endothelial cells synthesize and display heparin sulfate proteoglycans that have anticoagulant properties on their surface. Antithrombin, a small glycoprotein produced by the liver, binds to heparin sulfate proteoglycans and inactivates thrombin. Tissue factor pathway inhibitors (TFPIs) are synthesized by endothelial cells and bind to heparin sulfate proteoglycans. TFPIs inhibit the TF/FVIIa complex and prevent thrombin generation. In addition, endothelial cells synthesize and display thrombomodulin, which inhibits thrombin activity and stimulates the protein C–protein S anticoagulant axis [13].

Key processes of preventing platelet activation include the inhibition of thrombin activity, conversion of a platelet agonist ADP to AMP, and inhibition of the physical interaction between platelets and collagen (Figure 2) [13]. Endothelial cells prevent platelet activation by inhibiting thrombin activity and producing CD39/ecto-ADPase that depletes ADP. In addition, endothelial cells sequester von Willebrand factor (vWF), a glycoprotein that strengthens the interaction between platelets and the basement membrane by keeping it within the intracellular granules called Weibel–Palade bodies in endothelial cells. Endothelial cells produce NO and prostaglandin I_2_, which synergistically inhibit platelet adhesion and aggregation on endothelial cells.

The resting endothelium resists prolonged contact with circulating leucocytes that bathe the intimal surface by inhibiting the expression of adhesion molecules that mediate leucocyte attachment [13,17,18]. P-selectin, which is basally expressed in human endothelial cells, is sequestered into Weibel–Palade bodies along with vWF (Figure 2). Transcriptions of other adhesion molecules, including E-selectin, intracellular adhesion molecule-1, and vascular cell adhesion molecule-1, are also suppressed in resting endothelial cells. The basal NO production may contribute to the maintenance of endothelium quiescence through several mechanisms, including the reduction of P-selectin expression on the endothelial surface through inhibiting the fusion of Weibel–Palade bodies with the surface of the endothelial cell, inhibiting transcription of adhesion molecules, and inhibiting the expression of proinflammatory genes [19,20]. Shear stress produced by flowing blood is the principal signal for basal NO production by resting endothelial cells.

## 3. Possibility of Direct SARS-Cov-2 Infection of Endothelial Cells

ACE2, an integral transmembrane protein that is abundantly present in various human tissues, has been confirmed as the main entry receptor for SARS-CoV-2, as well as for SARS-CoV [21,22]. Therefore, the tropism and infectivity of SARS-CoV-2 depend primarily on the expression of ACE2. SARS-CoV-2 entry into host cells also depends on the priming of viral surface spike proteins by host cell proteases to facilitate viral entry. The spike protein has two major domains: S1 that possesses a receptor-binding site and S2 that contains a fusion peptide. Priming by transmembrane protease serine 2 (TMPRSS2) via cleavage of the viral spike protein at the S1/S2 and S2′ sites is essential for SARS-CoV-2 entry into host cells [23]. In addition to TMPRSS2, other host proteases such as cathepsin L, cathepsin B, and furin may be involved in the spike protein priming for facilitating SARS-CoV-2 entry into host cells [23,24,25]. Taken together, the expression of TMPRSS2 and the expression of other proteases, as well as the expression of ACE2, determine cell susceptibility to SARS-CoV-2 infection.

Whether endothelial cells are directly infected by SARS-CoV-2 remains a matter of controversy. ACE2 has been shown to be expressed on vascular endothelial cells [21,26]. In addition, recent in vitro experimental studies using cultured cells have shown that TMPRSS2 is expressed in human endothelial cells, including adult human lung microvascular endothelial cells, human umbilical vein endothelial cells, and blood outgrowth endothelial cells [27,28]. Although further studies are needed to investigate the physiological expression and function of TMPRSS2 in endothelial cells, these findings suggest that SARS-CoV-2 can directly infect human endothelial cells via the ACE2 pathway.

Indeed, coronavirus-like particles were found in endothelial cells in multiple organs including the lung, kidney, brain, and skin by using electron microscopy analyses in postmortem studies of COVID-19 cases. Ackermann et al. reported the presence of SARS-CoV-2 within the cell membrane in lung microvascular endothelial cells in patients who died from COVID-19 [29]. Dittmayer et al. presented electron microscopic images of SARS-CoV-2 in a lung endothelial cell [30]. Varga et al. reported the presence of viral particles in endothelial cells of the glomerular capillary loops in a male renal transplant recipient who died from multisystem organ failure after SARS-CoV-2 infection [31]. Bradley et al. and Menter et al. reported the presence of viral particles in kidney endothelial cells in patients who died from COVID-19 [32,33]. Paniz-Mondolfi et al. reported the presence of virus particles within cytoplasmic vacuoles in brain capillary endothelial cells in a male patient with COVID-19 who died from respiratory failure [34]. Colmenero et al. reported the presence of coronavirus-like particles in a capillary endothelial cell by examining a skin biopsy specimen from a pediatric patient with skin lesions clinically diagnosed as COVID-19-related chilblains [35]. They also reported that immunohistochemistry for SARS-CoV-2 spike protein was positive in capillary endothelial cells [35]. In addition to those electron microscopic analyses, Monteil et al. provided in situ evidence for the infection and replication of SARS-CoV-2 in endothelial cells by using engineered human capillary organoids with CD31^+^ endothelial lining established from induced pluripotent stem cells; quantitative real-time PCR analysis showed that SARS-CoV-2 RNA increased from day 3 to day 6 after primary SARS-CoV-2 exposure to the capillary organoids, indicating the infection and replication of SARS-CoV-2 in endothelial cells [36].

In contrast to those studies, endothelial cells derived from human pluripotent stem cells were shown to be resistant to SARS-CoV-2 infection [37]. In addition, several researchers have argued that virus-like structures in endothelial cells observed by electron microscopy are not SARS-CoV-2 particles but cellular structures, such as rough endoplasmic reticulum, multivesicular bodies, and clathrin-coated vesicles, and that other complementary techniques for viral detection, such as immuno-electron microscopy, immunohistochemistry, and in situ hybridization, should be applied since it is difficult to discriminate viral particles from cellular structures solely by morphological evidence [30,38,39,40,41]. In one autopsy study in which immunochemistry analysis of lung sections was performed in patients with COVID-19, no evidence of SARS-CoV-2 infection in pulmonary endothelial cells was found [42]. However, a recent autopsy case series of COVID-19 showed that SARS-CoV-2 RNA was localized by in situ hybridization within the endothelium and tunica media of vessels in multiple organs, including the lung, kidney, brain, heart, liver, and pancreas, providing important evidence of direct SARS-CoV-2 infection of vascular endothelial cells in multiple organs [43]. Taken together, although further studies are needed for confirmation, the results of studies indicate that SARS-CoV-2 may directly infect vascular endothelial cells in multiple organs. A combination of direct SARS-CoV-2 infection of endothelial cells and the endothelial cell response to the systemic inflammatory process associated with COVID-19 may synergistically mediate endothelial damage, leading to the exacerbation of thromboinflammation. SARS-CoV-2 replication and discharge from endothelial cells following viral infection may disable or destroy endothelial cells, contributing to further exacerbation of thromboinflammation in patients with COVID-19.

## 4. Downregulation of ACE2 Expression and Endothelial Dysfunction in COVID-19

**Figure 3:**
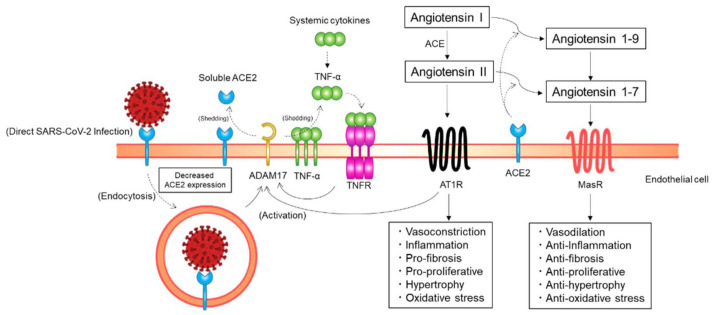
Putative mechanisms of a decrease in angiotensin-converting enzyme 2 (ACE2) expression on endothelial cells. ACE2 expression on endothelial cells may be downregulated through ACE2 endocytosis along with SARS-CoV-2 particles and ectodomain shedding of ACE2 by a disintegrin and metalloprotease 17 (ADAM17). AT1R, angiotensin II type 1 receptor; ACE, angiotensin-converting enzyme; MasR, Mas receptor; TNF, tumor necrosis factor; TNFR, TNF receptor.

ACE2, the main entry receptor for SARS-CoV-2, functions as a key counterregulatory enzyme of the renin–angiotensin system (RAS) mainly by degrading angiotensin I and angiotensin II to angiotensin 1–9 and angiotensin 1–7, respectively (Figure 3) [26,44]. Angiotensin II, the main effector peptide of the RAS, exerts its biological effects through binding to angiotensin II type 1 receptors (AT1Rs) and angiotensin II type 2 receptors (AT2Rs) [45]. The major pathophysiological actions of angiotensin II, including vasoconstriction, profibrosis, and proinflammation, are mediated via AT1Rs. Although AT2R-mediated biological actions of angiotensin II, such as vasorelaxation and natriuresis, are opposed to the AT1R-mediated deleterious actions, the exact physiological role of AT2Rs in humans is still unclear [26]. As opposed to angiotensin II, angiotensin 1–7 has cardiovascular protective effects, including vasodilatory, antifibrotic, and anti-inflammatory effects. NO release from the endothelium is increased by angiotensin 1–7 through post-transcriptional regulation of endothelial NO synthase (eNOS) induced by phosphorylation at Ser1177 and dephosphorylation at Thr495 via an Akt-dependent pathway [46]. Angiotensin 1–7 exerts its beneficial effects through binding to Mas receptors (MasRs) [47]. Therefore, the ACE2/angiotensin 1–7/MasR axis functions as a physiological antagonist that counterregulates the ACE/angiotensin II/AT1R axis. Taken together, ACE2 can exert its cardiovascular protective effects mainly by decreasing angiotensin II levels and increasing angiotensin 1–7 levels through the degradation of angiotensin II to angiotensin 1–7.

SARS-CoV-2 may not only gain initial entry into cells through ACE2 but also downregulate ACE2 expression in host cells (Figure 3). A previous study showed that the expression of ACE2 on the cell surface is substantially decreased after SARS-CoV spike protein binding to ACE2 [48]. In addition, continued infection and replication of human coronavirus-NL63 have been shown to downregulate membrane ACE2 expression, at least in in vitro cultured cells [49]. A similar process may occur for SARS-CoV-2; the initial engagement of SARS-CoV-2 spike proteins to ACE2 on the cell surface and subsequent viral replication in the host cell after SARS-CoV-2 entry may downregulate cellular ACE2 expression [44]. The membrane ACE2 expression may be decreased by endocytosis of ACE2 along with SARS-CoV-2 viral particles. ACE2 expression on the cell surface may be decreased through proteolysis and ectodomain shedding of ACE2 by activated a disintegrin and metalloprotease 17 (ADAM17), a type I transmembrane protein. The activity of AT1Rs is increased with an increase in angiotensin II levels at the cost of ACE2/angiotensin 1–7-driven pathways. Phosphorylation of ADAM17 is enhanced via the activated AT1R/p38 mitogen-activated protein kinase signaling pathway, and the catalytic activity of ADAM17 is subsequently upregulated, leading to a further decrease in ACE2 expression. The proteolytic cleavage of ACE2 by ADAM17 is increased through endosomal/lysosomal processing of endocytosed SARS-CoV-2 spike proteins, leading to a further decrease in ACE2 expression. ADAM17 is activated via the tumor necrosis factor (TNF)/TNF receptor pathway. Extracellular domain shedding of TNF-α is driven by ADAM17. Therefore, TNF receptors are activated by increased TNF-α production due to extracellular domain shedding of TNF-α by activated ADAM17 and systemic inflammation, which increases ADAM17 activity, leading to a further decrease in ACE2 expression. Taken together, ACE2 expression in host cells may be downregulated through ACE2 endocytosis along with SARS-CoV-2 particles and the ectodomain shedding of ACE2 by activated ADAM17 [44]. Although further studies are needed for confirmation in the clinical context, these findings indicate the possibility that ACE2 expression on the endothelial cell surface is decreased in patients with COVID-19.

Decreased ACE2 expression in endothelial cells may be pivotal in cardiovascular sequelae in patients with COVID-19. Conversions of angiotensin I and angiotensin II to angiotensin 1–9 and angiotensin 1–7 are impaired because of the decreased ACE2 expression following SARS-CoV-2 infection, resulting in a decrease in angiotensin 1–7 and an increase in angiotensin II. NO bioavailability may be decreased through a decrease in angiotensin 1–7-induced inhibition of eNOS activity, leading to endothelial dysfunction. Unabated angiotensin II activity may also be in part responsible for endothelial dysfunction and COVID-19-associated organ injury through promoting microvascular thrombosis, coagulopathy, inflammation, hypofibrinolysis, interleukin-6 (IL-6) production, and ROS production [50,51,52,53,54,55,56,57,58]. ACE2 is directly involved in the control of the bradykinin–kallikrein pathway. Bradykinin is processed by carboxypeptidases into Lys des-Arg9-BK and des-Arg9-BK, both of which are ligands for the endothelial bradykinin receptor B1 [59,60]. Decreased ACE2 activity fails to inactivate Lys des-Arg9-BK and des-Arg9-BK, which increases bradykinin receptor B1 activity, leading to the promotion of vascular permeability, inflammation, and oxidative stress [61,62]. Therefore, endothelial dysfunction and COVID-19-associated organ injury may in part be induced by increased angiotensin II at the cost of angiotensin 1–7 and increased bradykinin receptor B1 activity due to decreased ACE2 expression in endothelial cells.

## 5. Thromboinflammation and Endothelial Dysfunction In COVID-19

**Figure 4:**
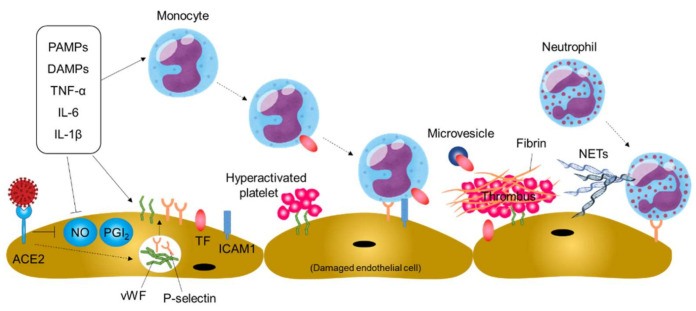
Thromboinflammation and endothelial dysfunction in COVID-19. Systemic inflammation and direct SARS-CoV-2 infection of endothelial cells may cause thromboinflammation, leading to thromboembolic events in patients with COVID-19. PAMPs, pathogen-associated molecular patterns; DAMPs, damage-associated molecular patterns; ACE2, angiotensin-converting enzyme 2; NO, nitric oxide; PGI_2_, prostaglandin I_2_; vWF, von Willebrand factor; TF, tissue factor; ICAM1, intercellular adhesion molecule-1; NETs, neutrophil extracellular traps.

An increase in TF expression in activated monocytes and endothelial cells is a pivotal step in the initiation and maintenance of thromboinflammation in blood vessels [63,64,65]. Blood monocytes are activated by circulating proinflammatory stimuli such as inflammatory cytokines, viral pathogen-associated molecular patterns, and damage-associated molecular patterns, leading to increased TF expression (Figure 4). In addition, TF-rich microvesicles are released from activated monocytes. Endothelial cells are damaged by SARS-CoV-2 infection and activated by proinflammatory molecules, leading to an increase in TF expression on endothelial cells. In addition, damaged and/or activated endothelial cells upregulate the expression of adhesion molecules and monocyte chemoattractants, promoting mobilization of monocytes to the damaged vessels [63]. As a result, the coagulation pathway is activated by increased TF expression, leading to thrombin generation, fibrin deposition, and blood clotting.

Damaged and/or activated endothelial cells produce neutrophil chemoattractants. In addition, the binding of thrombin to endothelial receptors upregulates the expression of P-selectin and vWF through mobilization of Weibel–Palade bodies within the endothelial cell, leading to neutrophil recruitment to the damaged vessels and platelet adhesion and activation on damaged vessels (Figure 4). The deposited platelets further promote neutrophil recruitment to the damaged vessels. The recruited neutrophils can release neutrophil extracellular traps (NETs), extracellular fibers composed of granule proteins and chromatin, for capturing viral particles and restricting viral dissemination in a process of NETosis [66,67]. However, NETs may aggravate damage to vascular endothelial cells and activate the coagulation pathway, leading to further platelet activation, fibrin deposition, and blood clotting.

During inflammation, major endogenous anticoagulation pathways, including TFPI, antithrombin, and protein C, are generally impaired, contributing to coagulation propagation.

While the prothrombotic risk in patients with COVID-19 is well recognized, the bleeding risk should not be ignored. A recent multicenter study showed that overall bleeding risk was 4.8% in hospitalized patients with COVID-19 and that the bleeding risk increased to 7.6% in critically ill patients [68], indicating a hyperfibrinolytic state in some groups of patients with COVID-19. The interplay between plasminogen activators and their principal inhibitor, plasminogen activator inhibitor-1 (PAI-1), plays a critical role in the regulation of fibrinolytic activity. Endothelial cells are a major source of tissue-type plasminogen activator (tPA) and PAI-1. Inflammatory cytokines may be a trigger of endothelial cell activation and promote local release of tPA and PAI-1. In addition, direct SARS-CoV-2 infection and destruction of endothelial cells may further promote the release of tPA and PAI-1 from the endothelium. Therefore, endothelial cells may play a pivotal role in the regulation of fibrinolytic activity in patients with COVID-19. Some studies have shown that plasma levels of both tPA and PAI-1 are elevated in hospitalized patients with COVID-19, indicating that effects of COVID-19 on fibrinolytic activity are variable and that a hyperfibrinolytic state exists in some patients with COVID-19 [69,70,71,72].

## 6. Endothelial Dysfunction in the Lung

The respiratory tract comprises distinct epithelial cell layers and vascular beds with a large surface area. Due to constant exposure to the outside environment, there is always a risk of acute lung infections, which can potentially develop into life-threatening conditions, especially when they lead to excessive recruitment and activation of inflammatory cells [73]. The lung harbors alveolar compartments with distinct vascular beds. The alveolar gas exchange surfaces of the lung contain the largest vascular bed in the body [73]. Because of the extremely delicate air–blood barrier for gas exchange, a very fine balance between protection and disease is maintained by resident immune cells in the lung for constant clearance of inhaled air particles and rapid response to pathogens through the highly coordinated innate and adaptive immune responses, which is critical for pathogen clearance. ACE2 is highly expressed on multiple types of respiratory tract epithelial cells, including alveolar type II epithelial cells in the lung parenchyma [24,74]. Therefore, the key targets for SARS-CoV-2 infection are epithelial cells of the bronchi and alveoli, which are constantly exposed to ambient air irritants and pathogens. Infected epithelial cells produce inflammatory mediators, leading to an immune response for viral clearance and the acquisition of antiviral immune memory. A failure to rapidly clear infection with SARS-CoV-2 can induce excessive uncontrolled inflammation and injury in the lung.

Hypoxic respiratory failure from ARDS is the leading cause of mortality in patients with COVID-19 [1]. Emerging evidence suggests that pulmonary vascular endothelial cells contribute to the initiation and propagation of ARDS by altering the integrity of the vessel barrier, promoting coagulation, and inducing vascular inflammation [75].

Vascular leakage and pulmonary edema observed in patients with COVID-19 are caused by multiple mechanisms. Direct SARS-CoV-2 infection of pulmonary endothelial cells causes endotheliitis characterized by endothelial cell dysfunction, lysis, and death [31]. Endothelial cells are damaged by histotoxic mediators, including ROS produced by activated neutrophils that are recruited to pulmonary endothelial cells. SARS-CoV-2 binding to ACE2 on the surface of pulmonary endothelial cells impairs the activity of ACE2, leading to activation of the kallikrein–bradykinin pathway and a subsequent increase in vascular permeability [13]. Immune cells, including monocytes and neutrophils; inflammatory cytokines, such as IL-1β, TNF, IL-6, and IL-8; and vasoactive molecules, such as thrombin, histamine, bradykinin, thromboxane A_2_, and vascular endothelial growth factor (VEGF), cause loosening of inter-endothelial junctions and enhancement of endothelial cell contractility, which pulls endothelial cells apart, leading to inter-endothelial gaps [13]. The glycocalyx is a gel-like layer that covers the surface of endothelial cells and plays a crucial role in the regulation of vascular permeability [76]. Cytokines, including IL-1β and TNF, upregulate glucuronidases that degrade the glycocalyx, leading to increased vascular permeability. Taken together, pulmonary vascular endothelial cell dysfunction is involved in the increase in pulmonary vascular permeability and vascular leakage, contributing to the initiation and propagation of ARDS in patients with COVID-19. Increased pulmonary vascular permeability may facilitate SARS-CoV-2 dissemination to the circulation [77].

A postmortem study in which seven lungs obtained from patients who died from COVID-19 were examined and compared with seven lungs obtained from patients who died from ARDS secondary to influenza showed that the number of alveolar capillary microthrombi was about 9 times larger in patients with COVID-19 than in patients with influenza [29]. Pulmonary vascular endothelial cell dysfunction may contribute to the formation of alveolar capillary microthrombi in patients with COVID-19. Pulmonary vascular endothelial cell damage induced by direct SARS-CoV-2 infection may be involved in coagulation propagation [29]. As a result of the disruption of pulmonary vascular integrity and endothelial cell death, the thrombogenic basement membrane is exposed in the lung, leading to activation of the coagulation cascade. In addition, expression of vWF, P-selectin, and fibrinogen is upregulated in pulmonary endothelial cells activated by inflammatory cytokines, including IL-1β and TNF, leading to attachment and activation of platelets [13]. The expression of TF is upregulated in activated pulmonary endothelial cells. TF expression on endothelial cells is upregulated by VEGF released from platelets. Increased TF expression leads to thrombin generation, fibrin formation, and blood clotting.

Inflammation is exacerbated by activated pulmonary endothelial cells. Direct SARS-CoV-2 infection of pulmonary endothelial cells may be involved in inflammation promotion. Increased expression of leukocyte adhesion molecules leads to the accumulation and extravasation of leukocytes, resulting in enhancement of lung tissue damage. In addition, denudation of the pulmonary blood vessels may lead to activation of the complement system and accumulation of neutrophils and monocytes, resulting in acceleration of the cytokine storm.

## 7. Endothelial Dysfunction in Extrapulmonary Blood Vessels

The respiratory tract is the primary site of COVID-19. Once SARS-CoV-2 particles spread beyond the pulmonary vascular barriers and are disseminated into non-respiratory blood vessels, SARS-CoV-2 may infect endothelial cells in other organs [78]. Endothelial cell injury in patients with COVID-19 is characterized by elevated levels of vWF and P-selectin and the presence of activated macrophages and neutrophils in multiple vascular beds including the heart, kidney, small intestine, and liver [78]. SARS-CoV-2 infection of endothelial cells in nonrespiratory organ may enhance the local activation of endothelial cells and upregulate the de novo transcription of adhesion molecules and inflammatory chemokines, which promotes the recruitment of neutrophils and monocytes, leading to a vicious cycle of local endothelial cell injury, additional leukocyte recruitment, and enhanced thromboinflammation. A cytokine storm originating in the lungs may amplify the systemic stimulation and damage to respiratory and nonrespiratory blood vessels. Endothelial damage might contribute to the high rates of cardiovascular complications in patients with COVID-19.

## 8. Conclusions

SARS-CoV-2 may directly infect endothelial cells via the ACE2 pathway. Cytokines and direct viral infection of endothelial cells may decrease ACE2 expression on endothelial cells, leading to an increase in angiotensin II levels and activation of the kallikrein–bradykinin pathway, which promotes coagulation, inflammation, oxidative stress, and permeability in patients with COVID-19. In addition, cytokines and direct viral infection of endothelial cells upregulate the expression of TF, adhesion molecules, and leukocyte chemoattractants in endothelial cells, promoting thromboinflammation. Therefore, the combination of a cytokine storm originating in the lungs and direct SARS-CoV-2 infection of endothelial cells may cause endothelial dysfunction, which contributes to the ischemic organ damage, thromboembolic complications, and severe disease outcomes in patients with COVID-19. However, it remains unclear when and how pulmonary vascular endothelial cells are targeted by SARS-CoV-2 and how the virus disseminates to extrapulmonary endothelial cells. Moreover, it is unknown whether SARS-CoV-2 infectivity to endothelial cells and the vascular complications in patients with severe COVID-19 are affected by chronic endothelial dysfunction induced by cardiovascular risk factors such as aging, hypertension, dyslipidemia, diabetes mellitus, and smoking. However, considering that patients with COVID-19 admitted to a hospital or an intensive care unit frequently present with conditions such as hypertension, diabetes mellitus, advanced age, and cardiovascular disease [79], prior endothelial dysfunction caused by cardiovascular risk factors may predispose to the development of vascular complications and severe disease outcomes in patients with COVID-19.

## Data Availability

Not applicable.
